# Clinical Features, Management, and Outcomes of Chest Trauma at a Tertiary-Care Centre in India: A Retrospective Observational Study

**DOI:** 10.1155/2021/8052586

**Published:** 2021-11-16

**Authors:** Bhupinder Singh Walia, Pankaj Dugg, Sanjeev Sharma

**Affiliations:** Department of General Surgery, Government Medical College, Amritsar, Punjab, India

## Abstract

**Introduction:**

Chest is one of the main sites of injuries in trauma being a part of the torso. Many important organs lie in rib cage. However, data on chest injuries are scarce.

**Methods:**

A retrospective study was carried out for chest trauma patients including polytrauma (*n* = 184) from hospital records for five years (2016–2020). Various parameters including demographic profile, mode of injury, management, and outcomes were studied.

**Results:**

Mean age of patients was 37 ± 16 years with a male to female ratio of 2.4 : 1. Road traffic injuries remained the most common cause of trauma followed by assaults. Most of the patients were managed conservatively (55.43%). Mortality was seen in only 1.63% patients.

**Conclusion:**

Young male patients are usually affected by trauma. Road traffic injuries are the commonest cause. However, most patients can be managed by conservative treatment and mortality is seen only in polytrauma patients in the present study.

## 1. Introduction

Traumatic injuries lead to 5.8 million deaths each year which accounts for 10% of the world's deaths. A trauma-related death is reported every 1.9 minutes in India [1]. Nearly 20 million are hospitalized every year due to injuries out which 1 million die due to trauma-related injuries [2].

Chest trauma accounts for about 10% of all trauma admissions and 25–50% of trauma deaths globally. The severity of chest trauma and associated comorbidities has been seen to determine the mortality due to chest trauma [3]. It has been reported that chest trauma is directly related to mortality in 25% of cases and has been a contributing factor in another 25%; however, a majority of patients (80%) can be managed with intercoastal chest tube drainage and appropriate analgesia and respiratory support [4].

The incidence of chest injuries has increased due to high-speed vehicular travels. It leads to life-threatening conditions such as tension pneumothorax, massive hemothorax, and injury to larger vessels [5]. The variation in the etiological pattern of chest trauma worldwide is attributed to many environmental and sociopolitical factors, but road traffic accidents (RTAs) remain the commonest cause of chest trauma in nonwar zones of the world [6].

Chest injuries have been associated with significant mortality. Although there is increased frequency of chest trauma patients in hospitals, data regarding the chest trauma are scarce [7].

A retrospective study is conducted on all chest trauma patients presented to our hospital between January 2016 and December 2020 to look for the management and outcome.

## 2. Methods

The study was carried out in a tertiary-care center. All patients admitted to department of surgery with chest trauma (including multiple trauma or exclusive chest injuries) were included in the study (*n* = 184). This study was based on retrospective observational analysis of data from hospital records from Jan 2016 to Dec 2020.

The analyzed data included demographic profile, mode of injury, types of chest injuries, management, and outcome.

Statistical analysis was conducted on SPSS software windows version 20. The ANOVA test was used for univariate analysis. The chi square test was used for comparison. A *P* value of <0.05 was considered significant. Microsoft excel worksheet version 2016 was used to calculate mean and standard deviation. Nominal data were presented as percentage.

## 3. Results

A total of 184 patients' data were included in the study who were admitted in the hospital with chest injury in last 5 years (Jan 2016 to Dec 2020).

Out of 184 patients, a majority of patients belonged to the age group of 21–30 years, i.e., 30.43%, followed by the age group of 41–50 years, i.e., 23.91%. The mean age of males and females was 36 ± 15 and 38 ± 18 years, respectively. Overall mean age was 37 ± 16 years (Table 1). The male to female ratio was 2.4 : 1.

Most of the injuries in this study were from road traffic accidents (*n* = 108, 58.69%). Assaults were the second most common mode of injury (*n* = 78, 42.39%). Other modes are less common (Table 2). A majority of patients had blunt trauma chest, and only 2% had penetrating trauma.

**Figure 1:**
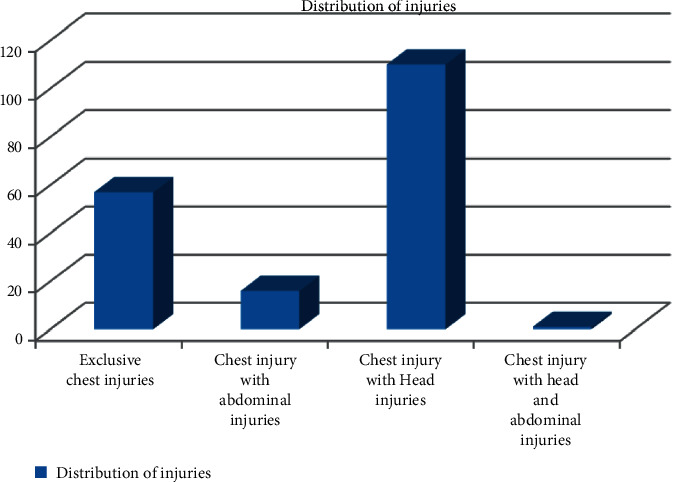
Distribution of injuries.

Among the type of injuries, abrasions were most common (*n* = 76) followed by bruises, hemothorax, and fracture ribs (Table 3). However, there were multiple findings in some cases. Exclusive chest injuries were seen in less number of patients (*n* = 57). A majority of patients had associated head injuries (*n* = 110). Associated abdominal injuries were seen in 16 patients, while one patient had both abdominal and head injuries (Figure 1).

Most of the cases were managed conservatively (*n* = 102, 55.43%). Only 68 patients required intercoastal chest tube drainage (36.96%) (Table 4). However, findings were not statistically significant as *P* value > .05.

Mortality is seen in 1.63% patients, while 79.35% (*n* = 146) were discharged in a satisfactory condition (Table 5). The three patients who expired had other injuries also. Two patients were of road traffic injuries having head injury with multiple rib fractures, while one patient was of railway track injury having head injury, abdominal injury (liver laceration), and flail chest.

Five patients were referred due to associated head injuries as the neurosurgeon was not available at our institute.

## 4. Discussion

Chest trauma contributes 10–15% of all trauma cases and is the root of 25–30% of all deaths due to trauma, making it an important health condition [8]. Most of the times, chest trauma is managed by general surgeons and thoracic surgeons are rarely a part of the team managing chest trauma [9]. Our institute is the referral centre of the region covering around five districts. Although there is no specific thoracic trauma unit, all the trauma cases are managed by general surgeons.

The mean age of patients affected by chest trauma were 37 ± 16 years, thus showing it affects younger age groups more commonly. The most common age group affected was 21–30 years (30.43%) followed by 41–50 years (23.91%). Kant et al. reported an average age of 36.25 years in patients of chest trauma [1]. Ibrahim et al. reported the maximum number of cases in the age group of 20–30 years with a decrease in the number of cases with an increase in age [8]. A similar trend is seen in our study after the age group of 41–50 years. Male population is most commonly affected in our study with a male to female ratio of 2.4 : 1. It was consistent with the study conducted by Liman et al. where males (70.6%) were predominantly affected as compared to female (29.4%) patients [10]. Male to female ratio in studies conducted by Kant et al. and Ekpe et al. was 3.54 : 1 and 4 : 1, respectively [1,3]. The reason for increased male population involved in trauma may be due to the reason that, in our country, most of the outdoor activities are carried out by male population but trends are changing in coming times and the findings in future may be different. Moreover, younger age groups have aggressive behavior and they are involved in dangerous driving and quarrels.

Road traffic injuries were the most common mode of injury (58.69%) in present study followed by assaults (42.39%). Although statistically findings are not significant (*P* < 0.05), assaults have become an important health problem in the society. As per the National Health Portal, road traffic injuries lead to five million deaths annually around the world. In India, one million die each year and around 20 million are hospitalized due to injuries. In 2015, the National Crime Records Bureau (NCRB) reported 413,457 deaths in road traffic injuries [11]. Kant et al. and Anisuzzaman et al. had similar findings with respect to mode of injury like the present study with road traffic injuries (63%) as the most common cause followed by assaults [1, 6]. Liman et al. and Mathangasinghe et al. reported road traffic injuries (67.79%) as the most common cause of chest trauma followed by falls [10, 12]. However, Ibrahim et al. reported assaults (42%) as the most common cause of chest trauma. The reason cited by them was characters of population activities in the area surrounding hospital [8]. Mode of trauma is blunt in majority cases with a ratio of 49 : 1 between blunt injuries and penetrating injuries in our study. Anisuzzaman et al. found only 0.8% cases of penetrating trauma in his study which is in accordance with the present study [6].

The most common injury reported in our study was abrasion (*n* = 76). Serious injuries such as hemothorax (*n* = 30), pneumothorax (*n* = 16), and flail chest (*n* = 8) associated with it were comparatively less. Choudhary et al. concluded in his study that most patients had soft-tissue injury and only intervention required in the study was intercoastal drainage [13]. In studies conducted by Kant et al. and Liman et al., the most common injury in the chest was rib fractures and flail chest [1, 10]. Ibrahim et al. reported open chest wall injuries as the most common presentation of chest injuries. The reason could be assaults contributing to the most common mode of injury [8]. Adegboye et al. in his study reported that blunt chest injuries lead to minor chest wall injuries (68%), major but stable chest wall injuries (7.6%), and flail chest injuries (10.8%). Thoracic injuries without fractures of bony chest wall occurred in 13.6% patients [14].

A majority of patients had associated head injuries in the present study with exclusive chest injuries seen in 30.97% patients only. Anisuzzaman et al. reported the most common associated injury was of the extremity followed by abdominal injury [6]. Mathangasinghe et al. also reported extremity injury as most common associated injury followed by head injury [12]. However, Choudhary et al. reported soft-tissue injuries in the form of lacerations as the common extrathoracic injuries [13].

Most of the patients were managed conservatively (55.43%), and only 68 (39.69%) patients required chest tube drainage. Most of the times, patients of chest trauma required conservative management only. No patient in the present study required thoracotomy. Kant et al. reported similar findings with 82% patients managed conservatively and 15% required chest tube drainage [1]. Kulshrestha et al. in their study concluded that most of chest trauma patients can be managed conservatively. In their study, only 18.32% patients required chest tube drainage and 2.6% required thoracotomy [7]. Liman reported that 25.7% patients required needle decompression out of which 2 patients required mediastinotomy. All patients of flail chest were managed in the intensive-care unit [10]. However, in study of Ibrahim et al., 58% patients required surgical interventions [8]. This could be due high number of open chest wall injuries.

In present study, a majority of patients (79.35%) were discharged in a satisfactory condition. Five patients required referral to a higher centre due to associated head injury. Mortality rate was 1.63%. Patients who expired had multiple injuries including abdominal and head injuries. All three patients belonged to higher age groups (>65 years). Mortality reported by Kulshreshtha et al. and Ibrahim et al. was 9.41% and 18%, respectively [7,8]. Mortality rate reported by Kant et al. is 2% [1]. Ekpe et al. reported that mortality in chest trauma is determined by associated extrathoracic organ injury, late presentation beyond 24 hour posttrauma, and severe chest injury with bilateral chest involvement [3].

Road traffic accidents are one of the increasing health problems in India and are becoming epidemic. Younger age groups are more commonly injured in traffic accidents. The causes are driving under the influence of alcohol and overspeeding [15]. Evidence-based interventions are required to improve road safety, enhance the involvement of the health system to deal with road injuries, and improve availability of quality actionable data. An improved plan for targeted interventions is required to achieve the Sustainable Development Goal (SDG) target by 2030 [16].

## 5. Conclusions

Road traffic injuries are the most common cause of chest trauma. However, assaults are also significantly increasing as an important cause of chest trauma. Mainly the young male population is being affected. Most of the times, chest trauma is managed conservatively and it has low mortality. Significant steps are required to prevent road traffic injuries and counselling sessions for younger adults involved in assaults.

## Figures and Tables

**Table 1:** Demographic profile (*n* = 184).

Age in years	Male	Female	Total
<10	2	2	4 (2.17%)
11–20	10	12	22 (11.96%)
21–30	46	10	56 (30.43%)
31–40	22	2	24 (13.04%)
41–50	30	14	44 (23.91%)
51–60	12	10	22 (11.96%)
>60	8	4	12 (6.52%)
Total	130	54	184
Mean ± SD (years)	36 ± 15	38 ± 18	37 ± 16

**Table 2:** Mode of injury.

Mode of injury	Male	Female	Total
Road traffic injury	72	26	108 (58.69%)
Assaults	54	24	78 (42.39%)
Falls	2	4	6 (3.26%)
Railway accidents	2	0	2 (1.09%)

**Table 3:** Type of chest injury.

Type of injury	No. of cases
Bruise	30
Abrasion	76
Laceration	14
Fractured ribs	30
Fractured clavicle	20
Flail chest	8
Pulmonary contusions	10
Pneumothorax	16
Hemothorax	30

**Table 4:** Management profile of patients.

Management/outcome	No. of cases
Suturing under LA	14 (7.61%)
Conservative	102 (55.43%)
Intercoastal chest tube drainage	68 (36.96%)
*P * **value**	0.686576

**Table 5:** Outcome of chest injury patients (*n* = 184).

Outcome	No. of cases
Discharged	146 (79.35%)
Referred	5 (2.72%)
Left against medical advise	30 (16.67%)
Expired	3 (1.63%)

## Data Availability

Data can be obtained from the corresponding author, e mail: dr_dugg@hotmail.com.
